# (2-Benzoyl­phen­yl)(2-meth­oxy-1-naphth­yl)methanone

**DOI:** 10.1107/S1600536811038049

**Published:** 2011-09-30

**Authors:** G. Jagadeesan, K. Sethusankar, R. Sivasakthikumaran, Arasambattu K. Mohanakrishnan

**Affiliations:** a Department of Physics, Dr MGR Educational and Research Institute, Dr MGR University, Chennai 600 095, India; b Department of Physics, RKM Vivekananda College (Autonomous), Chennai 600 004, India; c Department of Organic Chemistry, University of Madras, Maraimalai Campus, Chennai 600 025, India

## Abstract

In the title compound C_25_H_18_O_3_, the central benzene ring forms dihedral angles of 87.4 (5) and 85.4 (4)° with the phenyl ring and the naphthalene ring system, respectively. The carbonyl O atoms deviate significantly from the phenyl ring and the meth­oxy-substituted naphthalene ring system [by 0.508 (1) and 0.821 (1) Å, respectively]. The crystal packing is stabilized by C—H⋯O hydrogen bonds, which generate *C*(6) chains, and C—H⋯π inter­actions.

## Related literature

For chelating reagents of metallic systems, see: Liang *et al.* (2003 ▶). For the uses and biological importance of diketones, see: Bennett *et al.* (1999 ▶). For related structures, see: Tsumuki *et al.* (2011 ▶); Jagadeesan *et al.* (2011 ▶).
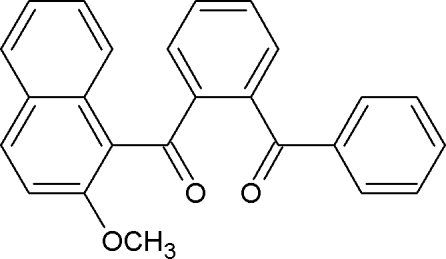


## Experimental

### 

#### Crystal data

C_25_H_18_O_3_
*M* *_r_* = 366.39
Monoclinic, 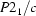

*a* = 15.0592 (7) Å
*b* = 7.6768 (3) Å
*c* = 16.9274 (8) Å
β = 106.137 (2)°
*V* = 1879.81 (15) Å^3^
*Z* = 4
Mo *K*α radiation
μ = 0.08 mm^−1^
*T* = 295 K
0.30 × 0.25 × 0.20 mm

#### Data collection

Bruker Kappa APEX II CCD diffractometer
22918 measured reflections
5339 independent reflections
3546 reflections with *I* > 2σ(*I*)
*R*_int_ = 0.026

#### Refinement

*R*[*F* ^2^ > 2σ(*F* ^2^)] = 0.046
*wR*(*F* ^2^) = 0.142
*S* = 1.00
5339 reflections
254 parameters
H-atom parameters constrained
Δρ_max_ = 0.28 e Å^−3^
Δρ_min_ = −0.17 e Å^−3^

### 

Data collection: *APEX2* (Bruker, 2008 ▶); cell refinement: *SAINT* (Bruker, 2008 ▶); data reduction: *SAINT*; program(s) used to solve structure: *SHELXS97* (Sheldrick, 2008 ▶); program(s) used to refine structure: *SHELXL97* (Sheldrick, 2008 ▶); molecular graphics: *ORTEP-3* (Farrugia, 1997 ▶); software used to prepare material for publication: *SHELXL97* and *PLATON* (Spek, 2009 ▶).

## Supplementary Material

Crystal structure: contains datablock(s) global, I. DOI: 10.1107/S1600536811038049/rk2301sup1.cif

Structure factors: contains datablock(s) I. DOI: 10.1107/S1600536811038049/rk2301Isup2.hkl

Supplementary material file. DOI: 10.1107/S1600536811038049/rk2301Isup3.cml

Additional supplementary materials:  crystallographic information; 3D view; checkCIF report

## Figures and Tables

**Table 1:** Hydrogen-bond geometry (Å, °) *Cg*1 is the centroid of the C8–C13 ring.

*D*—H⋯*A*	*D*—H	H⋯*A*	*D*⋯*A*	*D*—H⋯*A*
C10—H10⋯O1^i^	0.93	2.58	3.288 (2)	134
C19—H19⋯*Cg*1^ii^	0.93	2.77	3.585 (3)	147

Symmetry codes: (i) 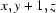; (ii) 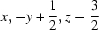.

**Fig. 1.**
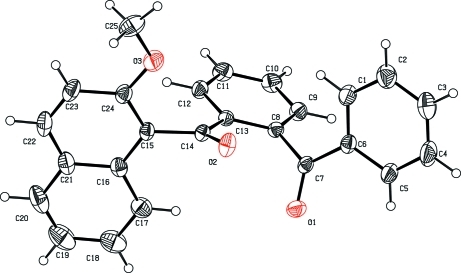
The molecular structure of the title compound with the atom numbering scheme. Displacement ellipsoids are drawn at the 30% probability level. H atoms are presented as a small spheres of arbitrary radius.

**Fig. 2.**
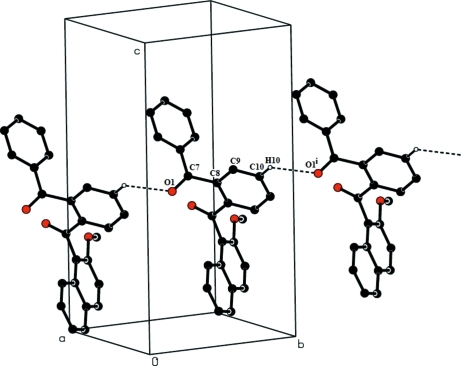
The packing arrangement of the title compound in the unit cell showing C10—H10···O1i intermolecular interactions. Symmetry code: (i) x, y+1, z.

## supplementary crystallographic information

### Comment

Diketones are employed as effective chelating reagents for a large number of metallic systems (Liang *et al.*, 2003). They are also popular in organic synthesis, for their applications in biology, medicine and also known to exhibit antioxidant, antitumour and antibacterial activities (Bennett *et al.*, 1999).

The molecular structure of the title compound C_25_H_18_O_3_, is shown at Fig. 1. The central phenyl ring (C8–C13) forms dihedral angles of 87.4 (5)° and 85.4 (4)° with the phenyl ring (C1–C6) and naphthalene moiety (C15–C24), respectively. The central phenyl ring (C8–C13) forms dihedral angles of 70.2 (5)° and 18.9 (5)° with the mean plane of the ketone groups (C6–C8/O1) and (C13–C15/O2), respectively. The dihedral angles between the naphthalene moiety (C15–C24) and phenyl ring (C1–C6) is 26.9 (4)°. The bond lengths and bond angles are normal and correspond to those observed in (2-benzoylphenyl)- (3,4-dimethylphenyl)-methanone (Jagadeesan *et al.*, 2011).

The two benzene rings (C15/16/C21–C24) and (C16–C21) are almost coplanar with dihedral angle of 1.54 (6)° between them. The atoms C25, O2 and O3 are having deviations of 0.213 (2)Å, 0.821 (1)Å and -0.013 (1)Å from the mean plane of the methoxy substituted naphthalene moiety (C15–C24), respectively. The atom O1 deviates by 0.508 (1)Å from the plane of the phenyl ring (C1–C6). The title compound exhibits the structural similarities with the reported related structures (Tsumuki *et al.*, 2011; Jagadeesan *et al.*, 2011).

In crystal packing, the molecules are linked *via* C10–H10···O1^i^ intermolecular interaction, which generates a *C*(6) chain. The crystal packing further stabilized by C19—H19···*Cg*1^ii^ interaction, where *Cg*1 is center of gravity of (C8–C13) ring (Table 1). Symmetry codes: (i) *x*, *y*+1, *z*; (ii) *x*, -*y*+1/2, *z*-3/2. The packing view of the compound is shown in (Fig. 2).

### Experimental

To a stirred suspension of 1-(2-methoxy-1-naphthyl)-3-phenyl-2-benzofuran (1 g, 3.22 mmol) in dry *THF* (20 ml), lead tetraaccetate (1.52 g, 3.42 mmol) was added and refluxed at 343 K for half an hour. The reaction mixture was then poured into water (200 ml) and extracted with ethyl acetate (2×20 ml), washed with brine solution and dried (Na_2_SO_4_). The removal of solvent *in vacuo* afforded crude product. The crude product upon crystallization from methanol furnished the tittle compound as a colourless solid.

### Refinement

Hydrogen atoms were placed in calculated positions with C—H = 0.93Å & 0.96Å and refined in the riding model with fixed isotropic displacement parameters: *U*_iso_(H) = 1.5*U*_eq_(C) for methyl group and *U*_iso_(H) = 1.2*U*_eq_(C) for aryl groups.

### Crystal data

**Table:**

C_25_H_18_O_3_	*F*(000) = 768
*M**_r_* = 366.39	*D*_x_ = 1.295 Mg m^−^^3^
Monoclinic, *P*2_1_/*c*	Mo *K*α radiation, λ = 0.71073 Å
Hall symbol: -P 2ybc	Cell parameters from 3546 reflections
*a* = 15.0592 (7) Å	θ = 1.4–29.8°
*b* = 7.6768 (3) Å	µ = 0.08 mm^−^^1^
*c* = 16.9274 (8) Å	*T* = 295 K
β = 106.137 (2)°	Block, colourless
*V* = 1879.81 (15) Å^3^	0.30 × 0.25 × 0.20 mm
*Z* = 4	

### Data collection

**Table:**

Bruker Kappa APEX II CCD diffractometer	3546 reflections with *I* > 2σ(*I*)
Radiation source: fine-focus sealed tube	*R*_int_ = 0.026
graphite	θ_max_ = 29.8°, θ_min_ = 2.5°
φ– and ω–scans	*h* = −21→20
22918 measured reflections	*k* = −7→10
5339 independent reflections	*l* = −23→23

### Refinement

**Table:**

Refinement on *F*^2^	Primary atom site location: structure-invariant direct methods
Least-squares matrix: full	Secondary atom site location: difference Fourier map
*R*[*F*^2^ > 2σ(*F*^2^)] = 0.046	Hydrogen site location: inferred from neighbouring sites
*wR*(*F*^2^) = 0.142	H-atom parameters constrained
*S* = 1.00	*w* = 1/[σ^2^(*F*_o_^2^) + (0.0646*P*)^2^ + 0.4893*P*] where *P* = (*F*_o_^2^ + 2*F*_c_^2^)/3
5339 reflections	(Δ/σ)_max_ < 0.001
254 parameters	Δρ_max_ = 0.28 e Å^−^^3^
0 restraints	Δρ_min_ = −0.17 e Å^−^^3^

### Special details

**Table:**

Geometry. All s.u.'s (except the s.u. in the dihedral angle between two l.s. planes) are estimated using the full covariance matrix. The cell s.u.'s are taken into account individually in the estimation of s.u.'s in distances, angles and torsion angles; correlations between s.u.'s in cell parameters are only used when they are defined by crystal symmetry. An approximate (isotropic) treatment of cell s.u.'s is used for estimating s.u.'s involving l.s. planes.
Refinement. Refinement of *F*^2^ against ALL reflections. The weighted *R*–factor *wR* and goodness of fit *S* are based on *F*^2^, conventional *R*–factors *R* are based on *F*, with *F* set to zero for negative *F*^2^. The threshold expression of *F*^2^ > σ(*F*^2^) is used only for calculating *R*–factors(gt) *etc*. and is not relevant to the choice of reflections for refinement. *R*–factors based on *F*^2^ are statistically about twice as large as those based on *F*, and *R*–factors based on ALL data will be even larger.

### Fractional atomic coordinates and isotropic or equivalent isotropic displacement parameters (Å^2^)

**Table:**

	*x*	*y*	*z*	*U*_iso_*/*U*_eq_	
C1	0.32655 (11)	0.5102 (2)	0.64285 (10)	0.0489 (4)	
H1	0.2815	0.5894	0.6159	0.059*	
C2	0.33289 (13)	0.4623 (3)	0.72296 (11)	0.0645 (5)	
H2	0.2915	0.5075	0.7494	0.077*	
C3	0.40028 (15)	0.3480 (3)	0.76349 (12)	0.0694 (5)	
H3	0.4044	0.3155	0.8173	0.083*	
C4	0.46149 (16)	0.2816 (3)	0.72472 (12)	0.0716 (6)	
H4	0.5076	0.2057	0.7528	0.086*	
C5	0.45535 (12)	0.3264 (2)	0.64411 (10)	0.0537 (4)	
H5	0.4969	0.2802	0.6181	0.064*	
C6	0.38683 (9)	0.44093 (17)	0.60238 (9)	0.0383 (3)	
C7	0.37750 (8)	0.48647 (16)	0.51530 (8)	0.0339 (3)	
C8	0.33986 (8)	0.66384 (16)	0.48624 (8)	0.0315 (3)	
C9	0.39631 (10)	0.80583 (18)	0.51630 (9)	0.0407 (3)	
H9	0.4518	0.7893	0.5567	0.049*	
C10	0.37067 (11)	0.97165 (19)	0.48663 (10)	0.0456 (3)	
H10	0.4091	1.0656	0.5071	0.055*	
C11	0.28879 (11)	0.99841 (18)	0.42712 (10)	0.0452 (3)	
H11	0.2719	1.1101	0.4073	0.054*	
C12	0.23162 (10)	0.85857 (17)	0.39690 (9)	0.0390 (3)	
H12	0.1763	0.8767	0.3565	0.047*	
C13	0.25602 (8)	0.69061 (15)	0.42638 (8)	0.0308 (3)	
C14	0.18976 (8)	0.54380 (17)	0.39884 (8)	0.0329 (3)	
C15	0.11451 (9)	0.57010 (17)	0.32057 (8)	0.0353 (3)	
C16	0.13622 (10)	0.57056 (18)	0.24428 (9)	0.0402 (3)	
C17	0.22628 (12)	0.5324 (2)	0.23795 (11)	0.0529 (4)	
H17	0.2735	0.5074	0.2852	0.063*	
C18	0.24417 (16)	0.5322 (3)	0.16352 (13)	0.0714 (5)	
H18	0.3033	0.5053	0.1603	0.086*	
C19	0.1752 (2)	0.5716 (3)	0.09233 (13)	0.0819 (7)	
H19	0.1888	0.5727	0.0420	0.098*	
C20	0.08820 (18)	0.6083 (3)	0.09543 (11)	0.0717 (6)	
H20	0.0429	0.6348	0.0471	0.086*	
C21	0.06502 (12)	0.6069 (2)	0.17118 (9)	0.0492 (4)	
C22	−0.02422 (13)	0.6403 (2)	0.17769 (11)	0.0572 (5)	
H22	−0.0710	0.6662	0.1303	0.069*	
C23	−0.04461 (11)	0.6362 (2)	0.25055 (11)	0.0533 (4)	
H23	−0.1048	0.6571	0.2526	0.064*	
C24	0.02565 (9)	0.60013 (19)	0.32376 (9)	0.0417 (3)	
C25	−0.08045 (13)	0.6050 (3)	0.40589 (14)	0.0703 (5)	
H25A	−0.1093	0.7120	0.3828	0.105*	
H25B	−0.0797	0.5981	0.4627	0.105*	
H25C	−0.1146	0.5083	0.3764	0.105*	
O1	0.40464 (7)	0.39130 (13)	0.46951 (6)	0.0455 (3)	
O2	0.19662 (7)	0.41118 (13)	0.43910 (7)	0.0480 (3)	
O3	0.01160 (7)	0.60000 (18)	0.39966 (7)	0.0601 (3)	

### Atomic displacement parameters (Å^2^)

**Table:**

	*U*^11^	*U*^22^	*U*^33^	*U*^12^	*U*^13^	*U*^23^
C1	0.0412 (8)	0.0565 (9)	0.0468 (9)	0.0060 (7)	0.0086 (6)	0.0080 (7)
C2	0.0571 (11)	0.0871 (14)	0.0491 (10)	0.0011 (10)	0.0148 (8)	0.0082 (9)
C3	0.0752 (13)	0.0791 (13)	0.0457 (10)	−0.0029 (11)	0.0034 (9)	0.0149 (9)
C4	0.0811 (14)	0.0612 (11)	0.0543 (11)	0.0194 (10)	−0.0114 (10)	0.0121 (9)
C5	0.0537 (9)	0.0470 (9)	0.0500 (9)	0.0148 (7)	−0.0029 (7)	0.0000 (7)
C6	0.0346 (7)	0.0327 (6)	0.0415 (7)	−0.0008 (5)	0.0004 (5)	0.0008 (5)
C7	0.0245 (6)	0.0307 (6)	0.0424 (7)	−0.0018 (5)	0.0023 (5)	−0.0020 (5)
C8	0.0296 (6)	0.0302 (6)	0.0345 (6)	−0.0002 (5)	0.0087 (5)	−0.0015 (5)
C9	0.0352 (7)	0.0374 (7)	0.0455 (8)	−0.0046 (5)	0.0043 (6)	−0.0055 (6)
C10	0.0493 (8)	0.0324 (7)	0.0544 (9)	−0.0093 (6)	0.0132 (7)	−0.0070 (6)
C11	0.0584 (9)	0.0273 (6)	0.0512 (9)	0.0015 (6)	0.0172 (7)	0.0035 (6)
C12	0.0416 (7)	0.0351 (7)	0.0373 (7)	0.0042 (5)	0.0058 (6)	0.0040 (5)
C13	0.0312 (6)	0.0292 (6)	0.0311 (6)	0.0004 (5)	0.0069 (5)	−0.0008 (5)
C14	0.0278 (6)	0.0347 (6)	0.0345 (7)	0.0002 (5)	0.0058 (5)	0.0010 (5)
C15	0.0315 (6)	0.0334 (6)	0.0357 (7)	−0.0017 (5)	0.0005 (5)	0.0007 (5)
C16	0.0460 (8)	0.0334 (7)	0.0373 (7)	−0.0054 (6)	0.0053 (6)	−0.0002 (5)
C17	0.0548 (9)	0.0566 (9)	0.0504 (9)	−0.0036 (8)	0.0199 (7)	−0.0007 (7)
C18	0.0841 (14)	0.0756 (13)	0.0664 (13)	−0.0067 (11)	0.0404 (11)	−0.0011 (10)
C19	0.119 (2)	0.0822 (15)	0.0550 (12)	−0.0156 (14)	0.0412 (13)	−0.0003 (10)
C20	0.1073 (17)	0.0608 (11)	0.0361 (9)	−0.0140 (11)	0.0019 (10)	0.0057 (8)
C21	0.0609 (10)	0.0378 (7)	0.0388 (8)	−0.0070 (7)	−0.0028 (7)	0.0028 (6)
C22	0.0588 (10)	0.0436 (8)	0.0500 (10)	−0.0019 (7)	−0.0167 (8)	0.0052 (7)
C23	0.0327 (7)	0.0482 (9)	0.0669 (11)	0.0017 (6)	−0.0063 (7)	0.0003 (7)
C24	0.0334 (7)	0.0407 (7)	0.0460 (8)	−0.0009 (6)	0.0027 (6)	−0.0014 (6)
C25	0.0454 (10)	0.0783 (13)	0.0950 (15)	0.0089 (9)	0.0326 (10)	0.0045 (11)
O1	0.0449 (6)	0.0386 (5)	0.0523 (6)	0.0062 (4)	0.0124 (5)	−0.0055 (4)
O2	0.0410 (6)	0.0397 (5)	0.0546 (6)	−0.0075 (4)	−0.0011 (5)	0.0132 (5)
O3	0.0362 (6)	0.0881 (9)	0.0573 (7)	0.0048 (6)	0.0153 (5)	−0.0010 (6)

### Geometric parameters (Å, °)

**Table:**

C1—C2	1.382 (2)	C14—O2	1.2135 (16)
C1—C6	1.386 (2)	C14—C15	1.4986 (17)
C1—H1	0.9300	C15—C24	1.3737 (19)
C2—C3	1.373 (3)	C15—C16	1.417 (2)
C2—H2	0.9300	C16—C17	1.420 (2)
C3—C4	1.370 (3)	C16—C21	1.422 (2)
C3—H3	0.9300	C17—C18	1.360 (2)
C4—C5	1.385 (3)	C17—H17	0.9300
C4—H4	0.9300	C18—C19	1.388 (3)
C5—C6	1.390 (2)	C18—H18	0.9300
C5—H5	0.9300	C19—C20	1.355 (3)
C6—C7	1.483 (2)	C19—H19	0.9300
C7—O1	1.2156 (16)	C20—C21	1.419 (3)
C7—C8	1.5041 (17)	C20—H20	0.9300
C8—C9	1.3891 (18)	C21—C22	1.402 (3)
C8—C13	1.3971 (18)	C22—C23	1.352 (3)
C9—C10	1.383 (2)	C22—H22	0.9300
C9—H9	0.9300	C23—C24	1.415 (2)
C10—C11	1.374 (2)	C23—H23	0.9300
C10—H10	0.9300	C24—O3	1.3589 (19)
C11—C12	1.382 (2)	C25—O3	1.420 (2)
C11—H11	0.9300	C25—H25A	0.9600
C12—C13	1.3945 (17)	C25—H25B	0.9600
C12—H12	0.9300	C25—H25C	0.9600
C13—C14	1.4915 (17)		
			
C2—C1—C6	120.42 (15)	O2—C14—C15	122.34 (11)
C2—C1—H1	119.8	C13—C14—C15	116.77 (11)
C6—C1—H1	119.8	C24—C15—C16	120.62 (12)
C3—C2—C1	120.02 (18)	C24—C15—C14	119.56 (12)
C3—C2—H2	120.0	C16—C15—C14	119.78 (12)
C1—C2—H2	120.0	C15—C16—C17	122.35 (13)
C4—C3—C2	120.05 (18)	C15—C16—C21	118.97 (14)
C4—C3—H3	120.0	C17—C16—C21	118.66 (14)
C2—C3—H3	120.0	C18—C17—C16	120.63 (17)
C3—C4—C5	120.67 (17)	C18—C17—H17	119.7
C3—C4—H4	119.7	C16—C17—H17	119.7
C5—C4—H4	119.7	C17—C18—C19	120.7 (2)
C4—C5—C6	119.65 (17)	C17—C18—H18	119.6
C4—C5—H5	120.2	C19—C18—H18	119.6
C6—C5—H5	120.2	C20—C19—C18	120.66 (19)
C1—C6—C5	119.15 (14)	C20—C19—H19	119.7
C1—C6—C7	120.43 (12)	C18—C19—H19	119.7
C5—C6—C7	120.41 (13)	C19—C20—C21	121.09 (18)
O1—C7—C6	122.44 (12)	C19—C20—H20	119.5
O1—C7—C8	119.89 (12)	C21—C20—H20	119.5
C6—C7—C8	117.48 (11)	C22—C21—C20	123.45 (16)
C9—C8—C13	119.21 (12)	C22—C21—C16	118.35 (15)
C9—C8—C7	117.00 (11)	C20—C21—C16	118.19 (18)
C13—C8—C7	123.56 (11)	C23—C22—C21	122.22 (14)
C10—C9—C8	120.56 (13)	C23—C22—H22	118.9
C10—C9—H9	119.7	C21—C22—H22	118.9
C8—C9—H9	119.7	C22—C23—C24	119.96 (15)
C11—C10—C9	120.46 (13)	C22—C23—H23	120.0
C11—C10—H10	119.8	C24—C23—H23	120.0
C9—C10—H10	119.8	O3—C24—C15	116.51 (12)
C10—C11—C12	119.67 (13)	O3—C24—C23	123.58 (14)
C10—C11—H11	120.2	C15—C24—C23	119.86 (15)
C12—C11—H11	120.2	O3—C25—H25A	109.5
C11—C12—C13	120.70 (13)	O3—C25—H25B	109.5
C11—C12—H12	119.6	H25A—C25—H25B	109.5
C13—C12—H12	119.6	O3—C25—H25C	109.5
C12—C13—C8	119.39 (11)	H25A—C25—H25C	109.5
C12—C13—C14	120.00 (11)	H25B—C25—H25C	109.5
C8—C13—C14	120.45 (11)	C24—O3—C25	118.82 (14)
O2—C14—C13	120.88 (11)		
			
C6—C1—C2—C3	1.2 (3)	O2—C14—C15—C24	73.13 (18)
C1—C2—C3—C4	0.2 (3)	C13—C14—C15—C24	−105.93 (14)
C2—C3—C4—C5	−1.1 (3)	O2—C14—C15—C16	−109.33 (16)
C3—C4—C5—C6	0.5 (3)	C13—C14—C15—C16	71.61 (16)
C2—C1—C6—C5	−1.8 (2)	C24—C15—C16—C17	−177.12 (14)
C2—C1—C6—C7	177.37 (15)	C14—C15—C16—C17	5.4 (2)
C4—C5—C6—C1	0.9 (2)	C24—C15—C16—C21	1.5 (2)
C4—C5—C6—C7	−178.24 (15)	C14—C15—C16—C21	−175.97 (12)
C1—C6—C7—O1	−154.41 (14)	C15—C16—C17—C18	179.22 (16)
C5—C6—C7—O1	24.7 (2)	C21—C16—C17—C18	0.6 (2)
C1—C6—C7—C8	30.61 (18)	C16—C17—C18—C19	0.9 (3)
C5—C6—C7—C8	−150.25 (13)	C17—C18—C19—C20	−1.1 (3)
O1—C7—C8—C9	−104.93 (15)	C18—C19—C20—C21	−0.3 (3)
C6—C7—C8—C9	70.18 (16)	C19—C20—C21—C22	−178.64 (18)
O1—C7—C8—C13	69.48 (17)	C19—C20—C21—C16	1.7 (3)
C6—C7—C8—C13	−115.41 (14)	C15—C16—C21—C22	−0.2 (2)
C13—C8—C9—C10	−0.9 (2)	C17—C16—C21—C22	178.51 (14)
C7—C8—C9—C10	173.74 (13)	C15—C16—C21—C20	179.46 (14)
C8—C9—C10—C11	0.2 (2)	C17—C16—C21—C20	−1.8 (2)
C9—C10—C11—C12	0.1 (2)	C20—C21—C22—C23	179.24 (16)
C10—C11—C12—C13	0.2 (2)	C16—C21—C22—C23	−1.1 (2)
C11—C12—C13—C8	−0.9 (2)	C21—C22—C23—C24	1.1 (2)
C11—C12—C13—C14	174.35 (13)	C16—C15—C24—O3	−178.97 (12)
C9—C8—C13—C12	1.28 (19)	C14—C15—C24—O3	−1.45 (19)
C7—C8—C13—C12	−173.02 (12)	C16—C15—C24—C23	−1.6 (2)
C9—C8—C13—C14	−174.00 (12)	C14—C15—C24—C23	175.93 (13)
C7—C8—C13—C14	11.71 (19)	C22—C23—C24—O3	177.46 (15)
C12—C13—C14—O2	−158.80 (14)	C22—C23—C24—C15	0.3 (2)
C8—C13—C14—O2	16.44 (19)	C15—C24—O3—C25	−170.97 (15)
C12—C13—C14—C15	20.27 (18)	C23—C24—O3—C25	11.8 (2)
C8—C13—C14—C15	−164.48 (12)		

### Hydrogen-bond geometry (Å, °)

**Table:**

Cg1 is the centroid of the C8–C13 ring.

**Table:**

*D*—H···*A*	*D*—H	H···*A*	*D*···*A*	*D*—H···*A*
C10—H10···O1^i^	0.93	2.58	3.288 (2)	134
C19—H19···Cg1^ii^	0.93	2.77	3.585 (3)	147

Symmetry codes: (i) *x*, *y*+1, *z*; (ii) *x*, −*y*+1/2, *z*−3/2.
